# Protocol: A systematic review and meta-analysis of the role of fetal and infantile environmental exposure in etiopathogenesis of infantile hypertrophic pyloric stenosis

**DOI:** 10.1371/journal.pone.0247003

**Published:** 2021-02-16

**Authors:** Ozlem Boybeyi-Turer, Hasan Tolga Çelik, Umut Ece Arslan, Tutku Soyer, Feridun Cahit Tanyel, Sibel Kiran

**Affiliations:** 1 Department of Pediatric Surgery, Hacettepe University Faculty of Medicine, Ankara, Turkey; 2 Department of Pediatrics Division of Neonatology, Hacettepe University Faculty of Medicine, Ankara, Turkey; 3 Department of Health Research, Hacettepe University Institute of Public Health, Ankara, Turkey; 4 Turkey Academy of Sciences, Ankara, Turkey; 5 Departments of Occupational Health and Safety, Hacettepe University Institute of Public Health, Ankara, Turkey; Universidade Federal dos Vales do Jequitinhonha e Mucuri, BRAZIL

## Abstract

Infantile hypertrophic pyloric stenosis (IHPS) is one of the hallmark pediatric surgical diseases. However, its etiology remains incompletely understood. By systematically reviewing the literature, we aim to clarify the effect of the effect of occupational and environmental factors and role of nitric oxide (NO) metabolism in the etiopathogenesis of IHPS. The systematic review is drafted with the Preferred Reporting Items for Systematic Reviews and Meta-Analysis statement (PRISMA) and the Meta-analysis of Observational Studies in Epidemiology (MOOSE). Systematic literature search will be performed for the period 2000 (Jan) to 2020 (Dec) in the databases: MEDLINE, EMBASE, PubMed. The systematic search will cover the literature in English and Turkish language and will be limited to studies on human subjects. Four investigators will independently search the databases (MEDLINE, EMBASE, PubMed) according to the defined search strategy. The full-text of the selected articles will be screened independently by four reviewers, against the inclusion criteria. Descriptive data will be extracted from each study regarding: study details, methods, participants, outcomes and calculations of association for potential further statistical analysis. If meta-analysis could not be undertaken, systematic approach to analyzing the findings of included multiple studies will be described. Heterogeneity will be assessed by quantifying the inconsistency across studies using I^2^ statistic. Statistical analysis will be performed using Comprehensive Meta-Analysis Version 3.0 software. The p values lower than 0.05 will be considered statistically significant for all analyses.

## Introduction

Infantile hypertrophic pyloric stenosis (IHPS) is one of the most frequent disorders requiring surgery in the first year of life [1, 2]. After the first description by Hirschsprung in 1888, the first successful surgical management was performed by Ramstedt in 1912 [3]. The clinical presentation, diagnosis and management are well established; including non-bilious projectile vomiting presented at 2–8 weeks of life, olive shaped palpable mass in the epigastrium, and splitting the pyloric muscle with pyloromyotomy as the first choice of management [3, 4].

Despite IHPS being one of the most frequently treated pediatric surgical conditions, the exact cause of the postnatal pyloric muscle thickening—characteristic of IHPS—is unclear. The incidence of IHPS has been reported as 2–4 cases per 1,000 live births with wide variation between worldwide locations, ethnic origins and seasons [1–3]. This variability in the incidence suggests possible environmental factors being involved in the etiology of IHPS. The incidence of IHPS has been reported to be increased among male infants [1–3], first-born children [4], infants fed with formula [4], and infants exposed to macrolides [5, 6]. There are only three meta-analyses in the literature regarding this issue. Murchison et al. [6] reported the first one and stated that there is a significant correlation between postnatal erythromycin exposure and IHPS. Zhu et al. [4] reported the second one and stated that first-born, cesarean section delivery, preterm birth, and bottle-feeding are associated with IHPS. Abdellatif et al. [5] reported the last one and stated that macrolide use during pregnancy and breastfeeding has no correlation with IHPS. However, the effect of the occupational and environmental exposures on etiopathogenesis of IHPS has not been reviewed before.

In the etiopathogenesis, several theories such defect in nitric oxide (NO) metabolism [7] and hyperacidity [3] in the stomach have been documented. NO is a well-known major inhibitory neurotransmitter in the gastrointestinal tract and its deficiency is suggested to cause hypertrophy in the pyloric muscle contributing IHPS etiopathogenesis [7–11]. However, the exact mechanism of NO reduction has not been clearly defined yet. There are also inconsistent results regarding possible effects of folic acid deficiency [12], maternal smoking [13], maternal hyperthyroidism, and nalidixic acid use [14], and paternal occupation [15]. Since these reports about the possible exposures in IHPS etiopathogenesis have not been reviewed before, we aimed to systematically review the literature. By systematically reviewing the literature, we aim to clarify the effect of environmental factors (except previous systematic review topics; erythromycin, macrolides and birth related factors) and the role of nitric oxide metabolism in IHPS origin. Once etiology and pathophysiology addressed accurately, the information will be helpful in identifying the causative risk factors and suggesting probable ways to prevent the disease.

## Materials and methods

The systematic review is drafted with the Preferred Reporting Items for Systematic Reviews and Meta-Analysis statement (PRISMA) [16], and the Meta-analysis of Observational Studies in Epidemiology [17]. The study is supported by Hacettepe University, Scientific Research Council (Grant number: 18121). The study is registered on PROSPERO- international prospective register system of systematic reviews (Registration number: **CRD42020152460**). The protocol began on 1 November 2019 and is registered on 22 February 2020. The study is expected to be completed 1 September 2021.

### Objectives

The objective of this study is to clarify the effect of environmental factors (except previous systematic review topics; erythromycin, macrolides and birth related factors) and the role of nitric oxide metabolism in IHPS origin. Once etiology and pathophysiology addressed accurately, the information will be helpful in identifying the causative risk factors and suggesting probable ways to prevent the disease.

Primary aims are:

- To examine effect of environmental exposure in the etiopathogenesis of IHPS.
- To determine additional exposures regarding maternal and fetal health that may have possible effect on IHPS etiopathogenesis.

### Review questions

- What is the role of fetal exposure to environmental factors in IHPS etiopathogenesis?
- What is the role of infantile exposure to environmental factors in IHPS etiopathogenesis?
- Is there a role of maternal or paternal occupational exposure in IHPS etiopathogenesis?
- Is there a role of nitric oxide metabolism alterations in IHPS etiopathogenesis?

### Patient and public involvement

Since this review will be based on published studies, there will be no patient or public involvement. Formal ethical approval is not required for this study. An institutional collaboration is approved by the Local Ethical Committee (11/12/2018-2230) revealing that this study does not need an ethical approval.

### Study design

The systematic review will consider the longitudinal observational studies including cohort studies. All other type of studies (Letters / Editorials, Qualitative studies, Therapy-Treatment studies, Prognosis studies, cross-sectional studies, Case reports, Case series and Gray literature) will be excluded.

#### Inclusion criteria

- The observational studies including cohort studies
- Articles published in English and Turkish
- Articles published between 2000 (Jan) to 2020 (Dec)
- Articles with subject of fetus, newborn, infants aged between 0 day to 12 weeks and diagnosed as IHPS

#### Exclusion criteria

- Studies other observational ones (Letters / Editorials, Qualitative studies, Therapy-Treatment studies, Prognosis studies, Cross-sectional studies, Case reports, Case series and Gray literature)
- Articles published in other languages
- Articles published before 2000 (Jan)
- The studies on older children and on animal studies

### Search methods for identification

#### Electronic searches

Systematic literature search will be performed for the period 2000 (Jan) to 2020 (Dec) in the databases: MEDLINE, EMBASE, PubMed. The systematic search will cover the literature in English and Turkish language and will be limited to studies on human subjects. The search strategy will consist of a combination of controlled search words, related medical subject heading (MeSH) terms and text words and search strings: terms related to IHPS, fetus and neonates, environmental exposure. The editorials, letter to editor, experimental studies, case reports and the gray literature will be excluded.

#### Search strategy

The search will be performed with the following subheading and related search words.

- ***Population (P)***: The systematic review will include the studies on fetus, newborn, infants aged between 0 day to 12 weeks and diagnosed as IHPS. The search strategy will include fetus, newborn, infant and synonyms and related mesh terms. The studies on older children and on animal studies will be excluded.
- ***Exposure (I/E)***: The systematic review will include the studies related to any kind of fetal and infantile exposure; mother related exposures (nutrition, diagnosis, medicine related), environmental and occupational exposures (parental occupations, hobbies, and home environment related exposures), perinatal exposures (procedure related exposures, medication, devices and so on). The previously reported factors (birth order, gender, feeding habits, macrolide exposure) will be excluded.
- ***Comparison (C)***: The comparator will be healthy controls or IHPS cases who did not exposed to none of above mentioned exposures.
- ***Outcomes (O)***: The main outcome is IHPS. IHPS diagnosis should depend on ultrasonography results and surgical outcome. The surgical outcome given by medical records or reported by physician or diagnosis made by imaging will be considered as valid diagnostic definition for the outcome measure. The primary aim of the study is to define the relationship between environmental exposures and IHPS.

#### Study selection

Four investigators (OBT, TS, HTC, SK) will independently search the databases (MEDLINE, EMBASE, PubMed) according to the defined search strategy. Each investigator will screen the titles and abstracts and select the articles meeting the inclusion criteria of the search strategy via using EndNote software. The researchers will pair two by two and check each other’s selection. The disagreements will resolve by discussion between two researchers, or the opinion of the third researcher will be taken if needed. The full-text of the selected articles will be reviewed by the researchers independently for data extraction. Differences among researchers will be resolved by discussion among all researchers and consultants.

#### Data extraction

The full-text of the selected articles will be screened independently by four reviewers, against the inclusion criteria. For studies that are not excluded on the basis of the title/abstract, full text papers will be requested and assessed by four reviewers. Any discrepancies will be resolved through discussion, and if required, a fifth reviewer. To record decisions, we will use MSExcell and EndNote softwares.

We will first validate our specially designed data extraction form developed in MSExcel with a random sample of ten included studies. Two review authors will extract quantitative data independently and then we will calculate Cohen’s Kappa to determine level of agreement. We will improve the form until sufficient validity is reached. Four reviewers will then perform actual data extraction. Each reviewer will be cross-checked with a random 20% sample of data extracted from the included studies.

Descriptive data will be extracted from each study regarding: Study details (date of study, title, author and research question); Methods (study design, exposure, primary outcome, potential confounders and any other outcomes); Participants (population demographics (age, gender, antecedents and co-morbidities), inclusion and exclusion criteria and participation rate); Outcomes (outcome name and definition, outcome type, how it is measured/reported, missing data and reasons for missing data). Moreover, from the studies reporting the results as calculations of association (e.g, risk ratios, rate ratios, odds ratios, and relative risks), using statistical modeling (e.g., multiple logistic regressions or multiple linear regressions) we will extract these data for potential further statistical analysis. MSExcell spreadsheet will be used to record data.

### Data analysis

The minimum number of studies for synthesis will be accepted as eight in order to calculate pooled estimate for any risk factor. The studies using IHPS for outcome and having exposure data will be included for quantitative analysis.

If meta-analysis could not be undertaken, systematic approach to analyzing the findings of included multiple studies will be described. We will create a narrative synthesis of the findings from the included studies, structured around the type of exposure, target population characteristics, and type of outcome. We will pool summary estimates in form of multiple logistic regression odds ratios whenever possible.

Regarding the meta-analysis, heterogeneity will be assessed by using the inconsistency Index, I^2^ statistic across studies. The heterogeneity value I^2^ can be identified as I^2^ = 0–25% no heterogeneity, 25–50% regarded as low, 50–75% as moderate, and 75–100% as high. If I^2^ is above 0.50, it will be accepted as there is a significant heterogeneity across studies. A random-effects model, using the DerSimonian and Laird method, will be used to calculate the pooled odds ratio when heterogeneity is found. Otherwise fixed-effects model using the Mantel-Haenszel method will be used. We will also perform subgroup analyses stratified by ethnicity (comparison of IHPS prevalence between different ethnic groups), smoking habits (comparison of smoker or non-smoker mothers), maternal medicine use, delivery type, and any other defined specific exposure, when sufficient data are available. We will use mean effects for comparisons of the different subgroups. We will conduct sensitivity analyses to determine a particular study that having heavily influenced on the summary effect. The funnel plot and Egger’s test will be used to examine the publication bias. In presence of significant Egger’s test and asymmetrical funnel plots, trim and fill analyses will be performed and adjusted effect sizes will be reported. Statistical analysis will be performed using Comprehensive Meta-Analysis Version 3.0 software. The p values lower than 0.05 will be considered statistically significant for all analyses.

### Quality assessment

Critical appraisal of the quality of included studies will be undertaken to avoid drawing unsupported, inappropriate or unwarranted conclusions. Appraisal will be documented and will be taken into account during analysis. The quality of studies meeting the inclusion criteria will be appraised independently by two reviewers according to the MEVORECH tool developed by Ijaz et al. [18] for assessing risk of bias in nonrandomized studies of exposure. Following their guidelines, for all included studies the reviewers will assess whether there are clear research questions, and the data collected address the research question. Next, the reviewers will assess the risk of bias for each study, considering seven domains through which bias might be introduced into a non-randomized study: confounding and selection of participants, biases due to deviations from intended interventions, missing data, measurement of outcomes, and selection of the reported result. For each domain, MEVORECH signaling questions will be answered to document the assessment and formulate judgments about the risk of bias for each domain, and an overall judgment on risk of bias for the outcome and result is in assessed. The studies having at least two high risk results in each domain of MEVORECH tool will be assessed as low quality. Any discrepancies between reviewers will be resolved through discussion, and if required, a third reviewer will be consulted.

## Conclusion

Infantile hypertrophic pyloric stenosis (IHPS) is one of the hallmark pediatric surgical diseases. However, its etiology remains incompletely understood. By systematically reviewing the literature, we aim to clarify the effect of environmental factors (except previous systematic review topics; erythromycin, macrolides and birth related factors) and the role of nitric oxide metabolism in IHPS origin. Once etiology and pathophysiology addressed accurately, the information will be helpful in identifying the causative risk factors and suggesting probable ways to prevent the disease.

## Supporting information

S1 Checklist Preferred reporting items for systematic review and meta-analysis protocols 2015 checklist: Recommended items to address in a systematic review protocol. (DOC)
